# Assessment of service refinement and its impact on repeat HIV testing by client's access to Australia's universal healthcare system: a retrospective cohort study

**DOI:** 10.1002/jia2.25353

**Published:** 2019-08-04

**Authors:** Kathleen E Ryan, Anna L Wilkinson, Jason Asselin, David P Leitinger, Peter Locke, Alisa Pedrana, Margaret Hellard, Mark Stoové

**Affiliations:** ^1^ Burnet Institute Melbourne Vic. Australia; ^2^ School of Public Health and Preventive Medicine Monash University Melbourne Vic. Australia; ^3^ Department of Infectious Diseases Alfred Health Melbourne Vic. Australia; ^4^ PRONTO! Thorne Harbour Health Melbourne Vic. Australia; ^5^ Department of Infectious Diseases Monash University and Alfred Health Melbourne Vic. Australia

**Keywords:** HIV infections, testing, MSM, sexually transmitted diseases, community based, transients and migrants delivery of health care

## Abstract

**Introduction:**

Achieving the virtual elimination of HIV requires equitable access to HIV prevention tools for all priority populations. Restricted access to healthcare means migrants face particular barriers to HIV prevention services. In February 2016, a peer‐led rapid HIV testing service for gay, bisexual and other men who have sex with men (gay and bisexual men, GBM) in Melbourne, Australia, introduced free sexually transmissible infection (STI) testing funded through Medicare (Australia's universal healthcare system). Medicare ineligible migrant clients were required to pay up to $158AUD for STI tests. We determined the uptake of STI testing and assessed the impact on repeat HIV testing among Medicare eligible and ineligible clients.

**Methods:**

All HIV tests conducted between August 2014 and March 2018 were included. We describe client characteristics, STI testing uptake and HIV/STI positivity among Medicare eligible and ineligible clients. Repeat HIV testing, assessed as the percentage of HIV tests with a return test within six months, was compared pre‐integration (August 2014‐June 2016) and post‐integration(July 2016‐March 2018) of STI testing using segmented linear regression of monthly aggregate data for Medicare eligible and ineligible clients.

**Results:**

Analyses included 9134 HIV tests among 4753 individuals. Medicare ineligible clients were younger (*p* < 0.01), and fewer reported previously testing for HIV (*p* < 0.01) and high HIV risk sexual behaviours. There was no difference in HIV positivity between the two groups (*p* = 0.09). STI testing uptake was significantly lower among Medicare ineligible clients (7.6%, 85.3%; *p* < 0.01). Following STI testing introduction there was an immediate increase in six‐month return HIV testing (6.4%; *p* = 0.02) and a significantly increasing rate of return HIV testing between July 2016 and March 2018 (0.5% per month; *p* < 0.01) among Medicare eligible clients but no immediate change in return testing (−0.9%; *p* = 0.7) or the rate of change in return testing between July 2016 and March 2018 (0.1% per month; *p* = 0.3) among Medicare ineligible clients. In March 2018, six‐month return HIV testing was 52.3% and 13.2% among Medicare eligible and ineligible clients respectively.

**Discussion:**

Improvements in return HIV testing observed among Medicare eligible clients did not extend to Medicare ineligible clients highlighting the impact of inequitable access to comprehensive sexual healthcare on test‐and‐treat approaches to HIV prevention.

## 1 Introduction

UNAIDS HIV cascade targets 1 that promote the control HIV epidemics through testing, diagnosis and treatment of people living with HIV (PLHIV) cannot be achieved without ensuring key populations at increased risk of infection have equitable access to healthcare. However, migrant populations represent an important part of local HIV epidemics in many parts of the world 2, 3, 4, their common exclusion from publicly subsidised healthcare and affordable access to HIV testing and treatment 5, 6 represents a challenge to HIV control. Australia has seen a recent divergence of the HIV epidemic among gay and bisexual men (GBM), with declining diagnoses among Australian‐born and increasing diagnoses among GBM born overseas 7, 8. Recent analyses describing the HIV risk profile of Asian‐born GBM point to the potential role of higher undiagnosed HIV prevalence among young Asian‐born GBM as a potential driver of new HIV infections 8.

PRONTO! is a peer‐led rapid point‐of‐care (RPOC) HIV testing service located in Melbourne, Australia that sees a high number of overseas born GBM relative to most local specialist HIV testing services. PRONTO! was implemented to reduce barriers to frequent HIV testing commonly reported by Australian GBM 9, 10. Evaluation of PRONTO! found that while features of the HIV testing service model (e.g. community‐based, peer‐led, RPOC test) were highly acceptable 11, 12, the lack of sexually transmissible infection (STI) testing introduced a significant barrier to returning to the service for HIV testing 12.

To facilitate more frequent HIV testing, PRONTO! underwent a number of changes in February 2016 that allowed for the introduction of STI testing funded through Medicare, Australia's universal healthcare system. Medicare rebates for clinical services can only be claimed by clinicians. The colocation of a trans and gender diverse clinic and a HIV pre‐exposure prophylaxis (PrEP) clinic at PRONTO! allowed for the registration of primary care doctors and the introduction of STI testing (*Chlamydia trachomatis* (chlamydia) and *Neisseria gonorrhoeae* (gonorrhoea) urethral, rectal and pharyngeal PCR, syphilis serology and parallel HIV serology) and treatment. SMS reminders were also introduced at this time to prompt clients to return for a routine HIV test or to close off a window period for a high‐risk event. All clients have access to free HIV RPOC tests through state government funding of PRONTO!, but only Medicare eligible clients can access free STI testing. Medicare eligible clients include Australian citizens, permanent residents and citizens of countries that Australia has a reciprocal healthcare agreement with 13. All other clients are required to pay up to $157AUD up front for comprehensive STI testing (HIV and syphilis serology, gonorrhoea and chlamydia oropharyngeal, rectal and urethral testing), which some can claim back from their health insurance provider.

We measured the uptake of STI testing at PRONTO! and its impact on six‐month return testing among clients eligible and ineligible for Medicare rebated STI testing.

## 2 Methods

The PRONTO! service model has been described previously in detail 14. Briefly, GBM peer staff deliver pre‐ and post‐test discussion and perform a Unigold HIV 1/2 RPOC test in accordance with manufacturer's instructions 15. Following delivery of a negative HIV RPOC test result, clients are recommended when to return for routine testing based on local testing guidelines 16 and/or to close off a window period and invited to receive an SMS to remind them to book this appointment.

In February 2016, comprehensive STI testing was implemented at PRONTO!. When clients opt to receive STI testing, peer staff collect serology for HIV and syphilis testing and the pharyngeal swab for chlamydia and gonorrhoea testing, and instruct clients in self‐collection of rectal and urine samples for chlamydia and gonorrhoea testing. Clients attending PRONTO! with STI symptoms are referred to a doctor or practice nurse and offered presumptive treatment if these staff are on site. Peer staff does not examine clients or prescribe medication for the treatment of STIs or HIV. All positive STI results are delivered via a phone call and clients are offered an appointment with a doctor on site for treatment.

### 2.1 Data collection and analysis

PRONTO! is a participating site in the Australian Collaboration for Co‐ordinated Enhanced Sentinel Surveillance (ACCESS) network, with all HIV and STI test results automatically extracted from the clinics Patient Management System to this system using GRAHNITE software 17, 18. Clients are invited to complete a behavioural survey in the waiting area prior to each consultation. Behavioural survey data were collected and managed using Redcap electronic data capture tools hosted at the Burnet Institute 19. A unique numeric identifier assigned to clients at their first appointment at PRONTO! links testing and behavioural survey data.

Analyses included all HIV tests conducted between August 2014 and March 2018. Clients using PrEP were excluded as three‐month appointments are required for ongoing monitoring and prescription collection (Australian guidelines require urine biochemistry be measured at baseline and six‐monthly thereafter, we thus identified PrEP use as an HIV test with a request for urine biochemistry, or any subsequent HIV test (315 individuals, 1435 HIV tests)) 20, 21. All test events excluded due to PrEP use were among Medicare eligible clients. Consistent with previous assessment of test frequency, tests within 30 days were considered a single testing episode and excluded 22.

We report the number of HIV tests, the number of individuals testing and demographic and behavioural characteristics for Medicare eligible and ineligible clients over the observation period. We describe the post‐STI implementation uptake of STI testing as the proportion of RPOC HIV tests accompanied by STI tests and report STI positivity for Medicare eligible and ineligible clients. Differences on these outcomes were assessed using Chi squared analyses.

We used segmented linear regression to assess changes in six‐month return testing, defined as the proportion of tests per month that occurred among clients who had a previous test within 182 days, overall and for Medicare eligible and ineligible clients. We tested for the structural break in return testing among all PRONTO! clients and found that, while STI testing was introduced in February 2016, the break occurred in June 2016. Segmented linear regression with Newey‐West standard errors estimated the change in the proportion of six‐month return testing between the pre‐intervention (August 2014‐June 2016) and post‐intervention (July 2016 – March 2018) periods. The regression model estimated the β0 (the intercept), the β1(the slope prior to the intervention), the β2 (the immediate change in outcome at intervention) and the β3 (the change in pre‐ and post‐intervention slope) and the post‐intervention slope (β1 + β 3).

Data were analysed using *Stata Statistical Software: Release 14* (StataCorp LP, College Station, TX, USA) and the cut‐off for statistical significance was *p* < 0.05 for all analyses. This project was approved by the Alfred Health Research Ethics Committee (project number 297/13). Survey completion was voluntary and the need for consent to be collected from individual patients was waived by the ethics committee 18.

## 3 Results

Between August 2014 and March 2018, 4753 clients attended PRONTO! and received 9234 HIV tests. Compared with Medicare eligible clients, Medicare ineligible clients were younger, less likely to report a HIV test within previous six months, and less likely to report a high number of anal sex partners, inconsistent condom use, group sex or drug use before or during sex. Despite less reporting frequent sex behaviours associated with HIV transmission, HIV positivity was similar among Medicare ineligible compared with ineligible clients (Table 1).

**Table 1:** Characteristics of Medicare eligible and ineligible clients testing for HIV at PRONTO!

	Medicare ineligible	Medicare eligible	*p*‐value
Individuals	1472	3281	
Tests	2044	7190	
Surveys completed	1397	5532	
Age^a^			
16 to 29	619 (66.6)	1084 (46.1)	
30 to 39	254 (27.3)	778 (33.1)	
40+	57 (6.1)	491 (20.9)	<0.01
Median (IQR)	27 (24 to 31)	31 (26 to 39)	<0.01
Gender^a^			
Cis‐male	879 (93.7)	2334 (97.9)	
Cis‐female	40 (4.3)	20 (0.8)	
Trans‐male	13 (1.4)	11 (0.5)	
Trans‐female	5 (0.5)	5 (0.2)	
Non‐binary/genderfluid	<5	14 (0.6)	<0.01
Ever tested for HIV^a^			
Yes	605 (67.7)	1906 (81.0)	
No	289 (32.3)	448 (19.0)	<0.01
Number of male anal sex partners^b^			
None	252 (19.8)	466 (8.7)	
One	289 (22.7)	898 (16.8)	
2 to 10	651 (51.2)	3406 (63.7)	
11+	80 (6.3)	578 (10.8)	<0.01
Regular sex partners^b^	738 (69.8)	3310 (67.0)	0.08
Inconsistent condom use with regular sex partner^b^^,^ c	308 (41.7)	1087 (32.8)	<0.01
Casual sex partner^b^	922 (87.1)	4397 (88.8)	0.11
Inconsistent condom use with casual sex partners^b^^,^ c	542 (58.8)	2230 (50.7)	<0.01
Group sex with two or more men^b^	215 (19.1)	1686 (32.5)	<0.01
Drug use before or during sex^b^^,^ d	389 (29.6)	2477 (47.7)	<0.01

a At first test during data collection period.

b Six‐month recall.

c Among those reporting regular or casual sex partner.

d Drug use includes any of amyl nitrate, cannabis, cocaine, methamphetamine, amphetamine, ecstasy, Viagra.

Uptake of STI testing was significantly greater among Medicare eligible compared with ineligible clients; 85% (n = 3934) of HIV tests among Medicare eligible clients and 8% of HIV tests among Medicare ineligible tests were accompanied by any test. STI positivity was 8% (n = 379) and 0.4% (n = 4) among Medicare eligible and ineligible clients respectively (Table 2).

**Table 2:** STI testing uptake and positivity Medicare eligible and ineligible clients testing for HIV at PRONTO!, February 2016 – March 2018

	Medicare ineligible	Medicare eligible	*p*‐value
HIV test	1128	4598	
Diagnosed with HIV	7 (0.6)	13 (0.3)	0.09
Tested for any STI	86 (7.6)	3934 (85.3)	<0.01
Diagnosed with any STI^a^	4 (0.4)	379 (8.2)	
Tested for syphilis	76 (6.5)	3765 (81.7)	<0.01
Diagnosed with active syphilis^a^	2 (0.2)	37 (0.8)	
Tested for *N. gonorrhoea*	70 (6.2)	3772 (81.8)	<0.01
Diagnosed with Gonorrhoea^a^	2 (2.9)	236 (6.3)	
Tested for *C. trachomatis*	70 (6.2)	3781 (82.2)	<0.01
Diagnosed with Chlamydia^a^	1 (1.4)	234 (6.2)	

a Statistical test not performed due to small cell size.

**Figure 1:**
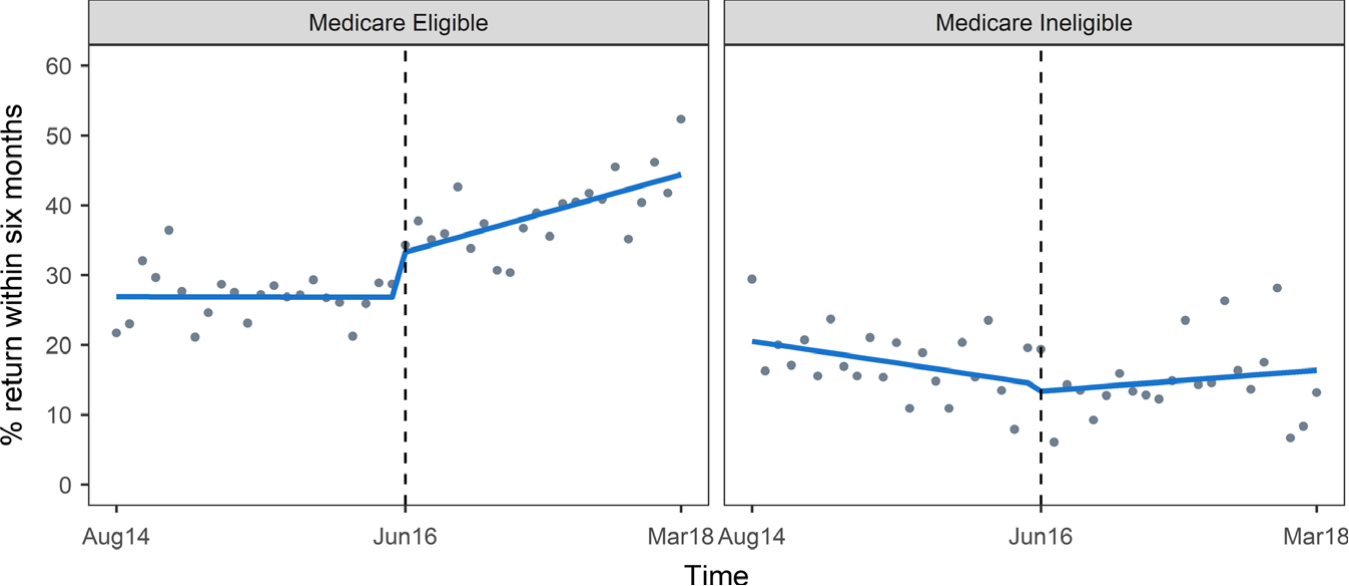
Segmented linear regression of six month return testing among Medicare eligible and ineligible clients attending PRONTO!, August 2014 – March 2018

Six‐month return testing among Medicare eligible clients was 26.9% in August 2014 and 26.1% in January 2016, with no meaningful change to the rate of return testing across this pre‐intervention period (β1 −0.004, 95% CI: −0.3% to 0.3%, *p* = 0.98). At interruption, six‐month return testing increased by an estimated 6.4% (95% CI: 1.2% to 11.7%, *p* = 0.02), from 28.7% in May 2016 to 34.3% in June 2016. By March 2018, six‐month return testing increased to 52.3%, representing an average monthly increase of 0.5% per month (*p* < 0.01) and a significant post‐intervention change in slope (β3 0.53, 95% CI: 0.11 to 0.95, *p* = 0.01) (Figure 1, Table 3).

**Table 3:** Segmented linear regression of six‐month return testing among Medicare ineligible and eligible clients attending PRONTO!, August 2014 – March 2018

	Medicare ineligible	*p*‐value	Medicare eligible	*p*‐value
	β (95% CI)		β (95% CI)	
β0 intercept	20.83 (16.96 to 24.69)	<0.01	26.93 (23.06 to 30.79)	<0.01
β1 pre‐slope	−0.28 (−0.58 to 0.01)	0.06	0.00 (−0.30 to 0.29)	0.98
β2 change at intervention	−0.93 (−6.27 to 4.41)	0.73	6.44 (1.22 to 11.66)	0.02
β3 change in slope	0.43 (0.01 to 0.84)	0.04	0.53 (0.11 to 0.95)	0.01
Post‐slope	0.14 (−0.15 to 0.44)	0.33	0.53 (0.24 to 0.82)	<0.01

Six‐month return testing among Medicare ineligible clients was 29.4% in August 2014 and 15.4% in January 2016, with no meaningful change to the rate of return testing across this pre‐intervention period (β1 −0.3%, 95% CI: −0.6% to 0.01%, *p* = 0.06). At interruption, six‐month return testing decreased by an estimated 0.9% (95% CI: −6.3% to 4.4%, *p* = 0.73), to 19.3% in June 2016. By March 2018, six‐month return testing was estimated at 13.2%, representing an average monthly increase of 0.4% per month (*p* = 0.3) and a significant post‐intervention change in slope (β3 0.43%, 95% CI: −0.01% to 0.84%, *p* = 0.04) (Figure 1, Table 3).

## 4 Discussion

There was high uptake of STI testing at this peer‐led RPOC HIV testing service among clients who could access STI testing at no cost (Medicare eligible), but minimal uptake by those who were required to pay (Medicare ineligible). Consistent with earlier evaluation findings, the introduction of STI testing had a significant effect on six‐month return testing for HIV, but again, this impact was most pronounced among Medicare eligible clients. These findings highlight the persistent inequitable access to healthcare for migrants in Australia and the subsequent barrier to Australia achieving its HIV elimination targets.

The primary aim of establishing PRONTO! was to reduce barriers to HIV testing, to facilitate high frequency HIV testing among GBM, and help reduce undiagnosed HIV and associated onward HIV transmission. After adding STI testing to the service model, PRONTO! achieved a six‐month return testing rate similar to that observed at an established local sexual health service and greater than that observed in high HIV caseload primary care clinics in Melbourne 22. However, PRONTO! clients who were born overseas and Medicare ineligible have derived little benefit from the introduction of STI testing because of a requirement to pay up‐front for STI tests. To obtain an Australian Student Visa a person must prove that they have private health insurance with an accredited provider. Uncertainty about what services are covered, privacy concerns and the need to pay up‐front and claim back expenses may limit use of private insurance when accessing sexual healthcare and these factors may have contributed to low uptake of STI testing at PRONTO!. This lack of access to subsidised healthcare for many migrants to Australia presents a challenge for Australia's HIV elimination efforts and may hamper efforts to achieve the virtual elimination of HIV.

A diverging HIV epidemic has been observed in Australia in recent years, with declining rate of diagnosis among Australian‐born GBM occurring at the same time as increasing diagnoses among Asian‐born GBM 7, 8. Approximately one‐third of the Medicare ineligible clients at PRONTO! were Asian‐born, over 70% of these Asian‐born clients were aged under 30 years and half had lived in Australia for less than three years (data not shown). These clients are similar to those being diagnosed with HIV at Melbourne Sexual Health Centre 8. With improvements in early post‐diagnosis HIV treatment and viral suppression in Australia, it is widely acknowledged that undiagnosed HIV is now the main driver of onward transmission 23. The lower levels of behavioural risk and STI positivity among Medicare ineligible clients but levels of HIV positivity comparable to Medicare eligible clients at PRONTO! and Melbourne Sexual Health Service 8 suggest a pool of undiagnosed HIV in migrants in Melbourne that is maintained through barriers to HIV testing and treatment. However, research into sexual networks and HIV genotyping is needed to confirm this hypothesis. In this context, there is an urgent need to address health systems and financial barriers to sexual health services for migrants at risk of HIV in Australia.

This study has some limitations. Repeat HIV testing was only assessed at PRONTO!. Missed intermittent HIV tests at other services, particularly those related to the need to access STI testing elsewhere in the pre‐implementation period, may have resulted in an under‐estimation of repeat HIV testing in this period. Second, we were not able to disaggregate the impact of the simultaneous changes that occurred at PRONTO! (STI testing, SMS reminders, Equinox clinic) in 2016. Finally, the segmented linear regression of aggregate monthly data estimates the association between the introduction of STI testing and six‐month return testing, but not causation.

## 5 Conclusions

The introduction of STI testing resulted in high uptake and increased HIV test frequency among clients who were Medicare eligible and could access testing at no cost. These benefits did not extend to clients ineligible for Medicare, with little uptake and minimal impact on HIV test frequency. These data highlight financial and other barriers that migrants in Australia face when accessing sexual healthcare. Activities that address these inequities in access to sexual healthcare include state funded STI testing for migrants, clarity on services covered by private insurers, and additional community models of sexual healthcare that are not Medicare funded, and are needed to achieve elimination targets.

## Competing interests

The authors have no competing interests to declare.

## Authors’ contributions

KR led the study and drafted the manuscript. AP, MH and MS conceived the study. AW and MS contributed to data analysis. All authors contributed to data collection and interpretation of findings. All authors read, commented and approved the final manuscript.
